# Atrial fibrillation in patients with left ventricular non-compaction: incidence and associations with stroke, heart failure, and mortality

**DOI:** 10.1093/cvr/cvag156

**Published:** 2026-07-09

**Authors:** Amir Askarinejad, Thomas F Lüscher, Gregory Y H Lip

**Affiliations:** Liverpool Centre for Cardiovascular Sciences at University of Liverpool, Liverpool John Moores University and Liverpool Heart & Chest Hospital, 6 W Derby St, Liverpool L7 8TX, UK; Center for Molecular Cardiology, University of Zürich, 4th Floor Wagistrasse, Schlieren 12 8952, Switzerland; Heart Division, Royal Brompton and Harefield Hospitals, Sydney Street, London SW3 6NP, UK; National Heart and Lung Institute, Imperial College London, Guy Scadding Building, Dovehouse St, London SW3 6LY, UK; School of Cardiovascular and Metabolic Medicine & Sciences, King's College London, Strand, London WC2R 2LS, UK; Liverpool Centre for Cardiovascular Sciences at University of Liverpool, Liverpool John Moores University and Liverpool Heart & Chest Hospital, 6 W Derby St, Liverpool L7 8TX, UK; Department of Clinical Medicine, Aalborg University, Fredrik Bajers Vej 7K, Aalborg 9220, Denmark; Department of Invasive Cardiology, Medical University of Bialystok, Jana Kilińskiego 1, Bialystok 15-089, Poland

**Keywords:** Atrial fibrillation, Left ventricular non-compaction, Stroke

## Abstract

**Aims:**

Left ventricular non-compaction (LVNC) is a genetic cardiomyopathy with presentations ranging from asymptomatic disease to heart failure, stroke, and sudden cardiac death. LVNC-related structural and functional abnormalities may increase atrial fibrillation (AF) risk, but data are limited. We assessed AF incidence in LVNC and compared outcomes in patients with vs. without AF.

**Methods and results:**

Two cohorts of patients were identified using the TriNetX platform: (i) patients with LVNC without a prior history of AF and (ii) patients with LVNC and AF. The primary objective was to assess the 3 year risk of a composite of all-cause mortality, stroke, acute myocardial infarction, and heart failure in the two cohorts. Hazard ratios (HRs) were derived from univariable Cox proportional models before and after propensity score matching (PSM). Matching was conducted using a greedy nearest-neighbour approach with a calliper of 0.1. Patients with LVNC and AF (*N* = 29 356) were older and exhibited a higher burden of comorbidities compared with LVNC patients without AF (*N* = 39 339). In this observational analysis, after PSM, AF in patients with LVNC was associated with higher risks of stroke [HR 1.466, 95% confidence interval (CI) 1.359–1.581], new-onset heart failure (HR 1.439, 95% CI 1.358–1.526), all-cause death (HR 1.255, 95% CI 1.200–1.312), and the composite outcome (HR 1.301, 95% CI 1.269–1.334) compared with LVNC patients without AF. The 1 year incidence of AF in patients with LVNC was 36 per 1000 person-years.

**Conclusion:**

In this observational cohort, LVNC was associated with a high incidence of AF, and the presence of AF was associated with higher risks of stroke, heart failure, and all-cause death. Further prospective studies are warranted to assess the prognostic impact of AF in patients with LVNC.

**Time for primary review: 66 days**

## 1. Introduction

Left ventricular non-compaction (LVNC) is a cardiomyopathy characterized by prominent trabeculations alternating with deep recesses between them, forming a bilayered myocardium consisting of a spongy inner endocardial layer and a thinner outer compact epicardial layer.^1^ The prevalence of LVNC ranges from 1.05% in healthy controls to 3.16% in athletes, and likely more in those of African descent, and up to 18.6% in pregnant individuals.^2^ Clinical phenotypes can range from mild and asymptomatic individuals to those manifesting severe heart failure, stroke, and sudden cardiac death.^3^ In addition, LVNC has been associated with cardiac arrhythmias including ventricular arrhythmias and atrial fibrillation (AF).^4^

AF, the most common arrhythmia worldwide, is associated with a high risk of mortality and morbidity, including stroke, heart failure, dementia, hospitalizations, as well as substantial healthcare costs.^5–7^ The structural and functional changes of the myocardium in LVNC put such patients at risk of arrhythmias, potentially including AF. In patients with both LVNC and AF, deep intertrabecular recesses, prominent trabeculations, impaired myocardial compaction, and the inherently elevated thromboembolic risk of AF together result in a markedly high-risk clinical profile; however, evidence regarding outcomes in this patient population, in whom LVNC and AF coexist, remains limited.^8^

In this study using a global federated health research network, we aimed to assess the incidence of AF in LVNC and secondly to compare clinical outcomes between LVNC patients with and without AF.

## 2. Methods

### 2.1 Study design

This retrospective observational study was performed using the TriNetX Research Network (TriNetX, Cambridge, MA, USA), a global federated health research network that delivers access to anonymized electronic medical records from over 130 healthcare organizations, consisting of academic medical centres, community hospitals, and outpatient clinics. The network comprises more than 300 million individuals, mainly in the United States.

Available data include demographics, diagnoses coded using the International Classification of Diseases, Ninth and Tenth Revisions, Clinical Modification (ICD-9-CM and ICD-10-CM) codes, and medications coded with Veterans Affairs codes or RxNorm classification systems, depending on the contributing institution. Additional information about the network is available online (https://trinetx.com/data-sets-analytics/).

TriNetX performs in accordance with the Health Insurance Portability and Accountability Act (HIPAA) and US federal law. All patient data are de-identified in accordance with HIPAA’s Privacy Rule, and no patient-level identifiers are accessible. Access to the data is supervised via formal data-sharing agreements with participating institutions. This study involved secondary analysis of de-identified human data from the TriNetX Research Network and was conducted in accordance with the principles of the Declaration of Helsinki. TriNetX provides access only to de-identified data; therefore, institutional review board approval and informed consent were not required. Further methodological information is presented in the Supplementary Material.

### 2.2 Cohort definition

The searches on the TriNetX online research platform were performed on 3 March 2026, using the US Collaborative Network. At the time of data extraction, 65 healthcare organizations across the United States had contributed eligible patient data.

Based on recorded ICD-10-CM diagnoses, two cohorts of patients were identified using the TriNetX platform: (i) patients with LVNC (ICD-10-CM: I42.5) occurring between 1 January 2014 and 1 January 2023, with exclusion of other cardiomyopathies including ischaemic cardiomyopathy (ICD-10-CM: I25.5), dilated cardiomyopathy (ICD-10-CM: I42.0), obstructive hypertrophic cardiomyopathy (ICD-10-CM: I42.1), other hypertrophic cardiomyopathy (ICD-10-CM: I42.2), and alcoholic cardiomyopathy (ICD-10-CM: I42.6) and without a prior history of AF (ICD-10-CM: I48) and (ii) patients with LVNC (ICD-10-CM: I42.5) occurring between 1 January 2014 and 1 January 2023, with the same exclusions for other cardiomyopathies (ICD-10-CM: I25.5, I42.0, I42.1, I42.2, and I42.6), and with AF (ICD-10-CM: I48).

Additionally, for sensitivity analyses, two other cohorts were defined by restricting the aforementioned cohorts to patients with cardiac imaging around the time of LVNC diagnosis. Specifically, two further sets of cohorts were defined: (i) patients with LVNC (ICD-10-CM: I42.5) occurring between 1 January 2014 and 1 January 2023, with exclusion of other cardiomyopathies including ischaemic cardiomyopathy (ICD-10-CM: I25.5), dilated cardiomyopathy (ICD-10-CM: I42.0), obstructive hypertrophic cardiomyopathy (ICD-10-CM: I42.1), other hypertrophic cardiomyopathy (ICD-10-CM: I42.2), and alcoholic cardiomyopathy (ICD-10-CM: I42.6), and additionally requiring transthoracic echocardiography [Current Procedural Terminology (CPT) 93306] within 6 months before to 6 months after the first recorded LVNC diagnosis and (ii) patients with LVNC (ICD-10-CM: I42.5) occurring between 1 January 2014 and 1 January 2023, with the same exclusions for other cardiomyopathies, and additionally requiring cardiac magnetic resonance imaging (CPT 75557 or CPT 75561) within 6 months before to 6 months after the first recorded LVNC diagnosis.

To provide an internal comparator analysis contextualizing whether the excess risk observed was specific to the LVNC phenotype rather than attributable to AF alone, two cohorts of patients with AF were identified using the TriNetX platform based on recorded ICD-10-CM diagnoses: (i) patients with LVNC (ICD-10-CM: I42.5) occurring between 1 January 2014 and 1 January 2023, with exclusion of other cardiomyopathies including ischaemic cardiomyopathy (ICD-10-CM: I25.5), dilated cardiomyopathy (ICD-10-CM: I42.0), obstructive hypertrophic cardiomyopathy (ICD-10-CM: I42.1), other hypertrophic cardiomyopathy (ICD-10-CM: I42.2), and alcoholic cardiomyopathy (ICD-10-CM: I42.6), and with AF (ICD-10-CM: I48) and (ii) patients with AF (ICD-10-CM: I48) occurring between 1 January 2014 and 1 January 2023, without a diagnosis of LVNC (ICD-10-CM: I42.5), and with the same exclusions for other cardiomyopathies (ICD-10-CM: I25.5, I42.0, I42.1, I42.2, I42.6).

For the negative control analysis, two cohorts of patients with AF were identified: (i) patients with LVNC (ICD-10-CM: I42.5) and AF (ICD-10-CM: I48), with exclusion of other cardiomyopathies including dilated cardiomyopathy (ICD-10-CM: I42.0), obstructive hypertrophic cardiomyopathy (ICD-10-CM: I42.1), other hypertrophic cardiomyopathy (ICD-10-CM: I42.2), ischaemic cardiomyopathy (ICD-10-CM: I25.5), alcoholic cardiomyopathy (ICD-10-CM: I42.6), and other restrictive cardiomyopathy (ICD-10-CM: I42.5, where applicable in the platform coding framework) and (ii) patients with AF (ICD-10-CM: I48) without documented cardiomyopathy, defined by exclusion of LVNC and the same cardiomyopathy diagnoses used above.

For the positive control analyses, two additional cohorts of patients with AF were identified: (i) patients with dilated cardiomyopathy (ICD-10-CM: I42.0) and AF (ICD-10-CM: I48) occurring between 1 January 2014 and 1 January 2023, with exclusion of other cardiomyopathies including obstructive hypertrophic cardiomyopathy (ICD-10-CM: I42.1), other hypertrophic cardiomyopathy (ICD-10-CM: I42.2), ischaemic cardiomyopathy (ICD-10-CM: I25.5), alcoholic cardiomyopathy (ICD-10-CM: I42.6), and LVNC and (ii) patients with hypertrophic cardiomyopathy, defined by obstructive hypertrophic cardiomyopathy (ICD-10-CM: I42.1), other hypertrophic cardiomyopathy (ICD-10-CM: I42.2), and hypertrophic cardiomyopathy (ICD-9-CM: 425.1), together with AF (ICD-10-CM: I48), occurring between 1 January 2014 and 1 January 2023, with exclusion of other cardiomyopathies including dilated cardiomyopathy (ICD-10-CM: I42.0), ischaemic cardiomyopathy (ICD-10-CM: I25.5), alcoholic cardiomyopathy (ICD-10-CM: I42.6), and LVNC (ICD-10-CM: I45.5).

### 2.3 Outcomes

The primary objective was to assess the 3 year risk of a composite of all-cause mortality, stroke, acute myocardial infarction, and heart failure in the two cohorts. The secondary objective was to assess all-cause mortality, stroke, acute myocardial infarction, and heart failure separately in the two cohorts. As an exploratory outcome, we assessed the 1 year incidence of AF in patients with LVNC. The outcome window period was from 1 day after index event till 1096 days after index event.

### 2.4 Statistical analysis

Baseline characteristics of patients with LVNC without a prior history of AF and patients with LVNC and AF were compared and balanced utilizing logistic regression with propensity score matching (PSM) at a 1:1 ratio. Matching was conducted using a greedy nearest-neighbour approach with a calliper of 0.1.

The following covariates were included in the PSM model: age at index, sex (female), race/ethnicity (White, Hispanic or Latino, Black or African American, and Asian), and baseline comorbidities including hypertensive diseases, ischaemic heart diseases, peripheral vascular diseases, cerebral infarction, diabetes mellitus, dyslipidaemia, heart failure, chronic obstructive pulmonary disease, chronic kidney disease (CKD), neoplasms, body mass index (BMI), and left ventricular ejection fraction (LVEF). In addition, baseline cardiovascular medications were included (beta-blockers, diuretics, antiarrhythmics, calcium channel blockers, angiotensin-converting enzyme (ACE) inhibitors, and anticoagulants). Absolute standardized differences (ASDs) were used to evaluate covariate balance between groups, with values <0.1 considered indicative of sufficient balance.

Univariable Cox proportional hazards models, before and after PSM, were used to calculate hazard ratios (HRs) and 95% confidence intervals (95% CIs) for the risk of adverse events. Adjusted multivariable Cox proportional hazards model was performed using the same covariates used for PSM.

To further characterize treatment patterns in patients with LVNC and AF, we performed a descriptive treatment pathways analysis within TriNetX. The index date was defined as the first date on which the cohort criteria were met. Treatments were assessed from the index date through 1095 follow-up days. The selected treatment categories were beta-blockers/related agents (CV 100), anticoagulants (BL 110), antiarrhythmic drugs (CV300), and catheter ablation of AF (CPT 93656). A line of treatment was defined as any treatment occurring within 1 day, and a line ended when a new treatment appeared after the first day, when the patient died, when the medical record ended, or when the analysis window ended.

Pre-specified subgroup analyses explored whether associations varied across patient characteristics: age ≥75 years, female sex, AF type, CKD, diabetes, and hypertension. For each subgroup, we repeated PSM and survival analyses using identical matching specifications and statistical approaches as in the primary analysis. Differences in HRs across subgroups were assessed for statistical significance using the *Z*-test.^9^

### 2.5 Sensitivity analyses

A sensitivity analyses were utilized to assess outcomes occurring between 31 and 1126 days after the index event, thereby excluding events during 1 month after index event and focusing on a more stable long-term risk period. Two further sensitivity analyses were conducted in cohorts restricted to patients who underwent echocardiography or cardiac magnetic resonance imaging within 6 months before or after the diagnosis of LVNC. In addition, an external sensitivity analysis was performed using data from the Global Collaborative Network rather than the US Collaborative Network. In addition, a further sensitivity analysis excluded patients who died during the study period to minimize the potential competing effect of death on the assessment of non-fatal outcomes.

All statistical analyses were performed using the TriNetX analytics platform, which integrates R (v3.2–3) and Python 3.7 libraries (scikit-learn for logistic regression; survival for Cox models). Missing data were not imputed. All tests were two-tailed, and statistical significance was defined as *P* < 0.05.

## 3. Results

### 3.1 Baseline characteristics

The final cohorts included 29,356 patients with LVNC and AF (mean age 69.1 ± 13.5 years; 38.2% female) and 39 339 patients with LVNC without AF (mean age 57.4 ± 17.6 years; 46.8% female) (*Table 1*). The study flow chart is shown in Supplementary material online, *Figure S1*.

**Table 1:** Baseline characteristics before and after PSM

Characteristic	Before PSM				After PSM			
	LVNC with AF (*N* = 29 356)	LVNC without AF (*N* = 39 339)	*P*-value	ASD	LVNC with AF (*N* = 19 615)	LVNC without AF (*N* = 19 615)	*P*-value	ASD
Age at index, years	69.1 ± 13.5	57.4 ± 17.6	<0.001	0.745	65.7 ± 13.8	65.8 ± 13.7	0.294	0.011
Female	11 209 (38.2%)	18 427 (46.8%)	<0.001	0.176	8007 (40.8%)	7861 (40.1%)	0.133	0.015
White	21 957 (74.8%)	25 151 (63.9%)	<0.001	0.237	13 803 (70.4%)	13 873 (70.7%)	0.438	0.008
Hispanic or Latino	1043 (3.6%)	2283 (5.8%)	<0.001	0.107	834 (4.3%)	813 (4.1%)	0.597	0.005
Black or African American	5024 (17.1%)	9685 (24.6%)	<0.001	0.185	4008 (20.4%)	3972 (20.2%)	0.652	0.005
Asian	373 (1.3%)	650 (1.7%)	<0.001	0.032	268 (1.4%)	280 (1.4%)	0.606	0.005
Hypertensive diseases	19 536 (66.5%)	19 085 (48.5%)	<0.001	0.371	11 702 (59.7%)	11 375 (58.0%)	0.001	0.034
Ischaemic heart diseases	12 595 (42.9%)	10 583 (26.9%)	<0.001	0.341	7145 (36.4%)	7011 (35.7%)	0.159	0.014
Peripheral vascular disease	2922 (10.0%)	2067 (5.3%)	<0.001	0.178	1544 (7.9%)	1479 (7.5%)	0.218	0.012
Cerebral infarction	2519 (8.6%)	1536 (3.9%)	<0.001	0.194	1235 (6.3%)	1163 (5.9%)	0.129	0.015
Diabetes mellitus	8261 (28.1%)	8058 (20.5%)	<0.001	0.179	5065 (25.8%)	4875 (24.9%)	0.027	0.022
Dyslipidaemia	14 778 (50.3%)	13 414 (34.1%)	<0.001	0.333	8574 (43.7%)	8295 (42.3%)	0.004	0.029
Heart failure	15 768 (53.7%)	12 078 (30.7%)	<0.001	0.479	8461 (43.1%)	8268 (42.2%)	0.049	0.020
Chronic obstructive pulmonary disease	4831 (16.5%)	3566 (9.1%)	<0.001	0.223	2692 (13.7%)	2592 (13.2%)	0.139	0.015
CKD	7411 (25.2%)	5550 (14.1%)	<0.001	0.283	3950 (20.1%)	3812 (19.4%)	0.080	0.018
Neoplasms	9357 (31.9%)	9581 (24.4%)	<0.001	0.168	5584 (28.5%)	5446 (27.8%)	0.121	0.016
Beta-blockers/related	19 768 (67.3%)	17 707 (45.0%)	<0.001	0.462	11 262 (57.4%)	10 944 (55.8%)	0.001	0.033
Diuretics	17 519 (59.7%)	15 116 (38.4%)	<0.001	0.435	9691 (49.4%)	9490 (48.4%)	0.042	0.021
Antiarrhythmics	13 695 (46.7%)	10 055 (25.6%)	<0.001	0.450	7163 (36.5%)	6954 (35.5%)	0.028	0.022
Calcium channel blockers	10 474 (35.7%)	7646 (19.4%)	<0.001	0.370	5400 (27.5%)	5262 (26.8%)	0.117	0.016
ACE inhibitors	12 476 (42.5%)	12 974 (33.0%)	<0.001	0.197	7641 (39.0%)	7415 (37.8%)	0.019	0.024
Anticoagulants	18 548 (63.2%)	11 968 (30.4%)	<0.001	0.695	9453 (48.2%)	9296 (47.4%)	0.113	0.016
LVEF, %	46.3 ± 15.9	45.1 ± 15.4	0.050	0.073	46.4 ± 15.6	45.4 ± 15.5	0.168	0.065
BMI, kg/m^2^	29.9 ± 7.7	29.7 ± 8.1	<0.001	0.034	30.6 ± 8.1	29.2 ± 7.5	<0.001	0.170

Observations represent unique patients; there were no technical replicates. Before PSM, the LVNC with AF and LVNC without AF cohorts included 29,356 and 39,339 patients, respectively. After PSM, 19 615 patients remained in each group. Categorical variables are presented as *n* (%) and were compared using a two-sided χ^2^ test. Continuous variables are presented as mean ± SD and were compared using a two-sided Student’s *t*-test. *P*-values are shown in the table.

Before PSM, patients with LVNC and AF were older and exhibited a higher burden of comorbidities compared with patients with LVNC without AF (*Table 1*). For instance, hypertension, diabetes mellitus, ischaemic heart disease, and heart failure were all more common among patients with LVNC and AF compared with LVNC patients without AF (*Table 1*). After PSM, demographics and cardiovascular comorbidities were well balanced between groups, although some residual imbalance remained for BMI (*Table 1*).

### 3.2 Treatment pathway in cohort of patients with LVNC and AF

In the treatment pathways cohort of 25 557 patients with LVNC and AF, beta-blockers/related agents were the most frequently observed treatment category on pathway, identified in 11 884 patients (46.5%), followed by anticoagulants in 11 024 (43.1%), beta-blocker plus anticoagulant combinations in 10 708 (41.9%), and antiarrhythmic drugs in 7719 (30.2%) (see Supplementary material online, *Figure S2A* and *Table S2*). A triple regimen consisting of beta-blockers, antiarrhythmic drugs, and anticoagulants was also common, occurring in 6190 patients (24.2%), while antiarrhythmic plus anticoagulant combinations were observed in 4982 patients (19.5%). Across lines of treatment, beta-blockers and anticoagulants remained the dominant treatment classes. In Line 1, beta-blockers were present in 28.1% of patients and anticoagulants in 24.9%, while beta-blocker plus anticoagulant combinations accounted for 19.0% and antiarrhythmic drugs for 11.2% (see Supplementary material online, *Figure S2B* and *Table S2*).

### 3.3 Survival analysis

#### 3.3.1 Incidence of new-onset AF in patients with LVNC

**Figure 1:**
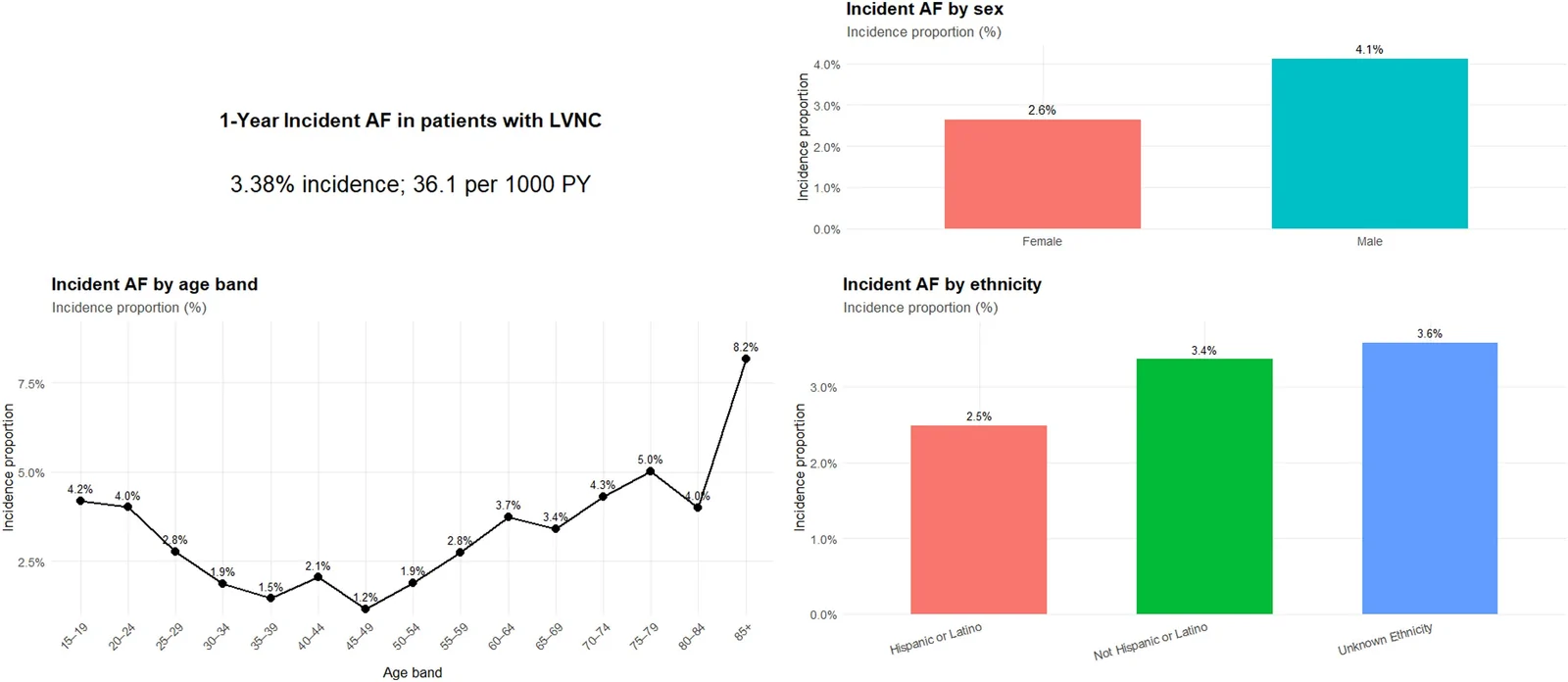
One year incidence of AF in patients with LVNC by sex, age, and ethnicity. Observations represent unique patients (biological replicates); there were no technical replicates. Incidence is reported as events per 1000 person-years over 1 year follow-up.

The 1 year incidence of AF in patients with LVNC was 36.1 per 1000 person-years (*Figure 1*) and was higher among older male patients and those who were not of Hispanic or Latino ethnicity (see Supplementary material online, *Table S1*).

#### 3.3.2 Follow-up duration and 3 year crude incidence of adverse outcomes

Before PSM, after a mean follow-up of 783.9 days [standard deviation (SD) 420.4] in the LVNC with AF cohort and 833.8 days (SD 407.1) in the LVNC without AF cohort [median 1095 days in both, inter-quartile range (IQR) 720 in LVNC with AF and 548 in LVNC without AF], the 3 year incidences were as follows: stroke in 4526 patients overall [2662 (9.1%) vs. 1864 (4.7%)], all-cause death in 12 641 patients overall [7385 (25.2%) vs. 5256 (13.4%)], acute myocardial infarction in 5500 patients overall [2761 (9.4%) vs. 2739 (7.0%)], and new-onset heart failure, in 7594 patients overall [3121 (33.1%) vs. 4473 (20.4%)] (*Table 2*).

**Table 2:** Three year crude incidence of outcomes before and after PSM

	Before PSM		After PSM	
Outcome	LVNC with AF (*n* = 29 356)	LVNC without AF (*n* = 39 339)	LVNC with AF (*n* = 19 615)	LVNC without AF (*n* = 19 615)
Stroke	2662 (9.1%)	1864 (4.7%)	1632 (8.3%)	1135 (5.8%)
All-cause death	7385 (25.2%)	5256 (13.4%)	4321 (22.0%)	3492 (17.8%)
Acute myocardial infarction	2761 (9.4%)	2739 (7.0%)	1701 (8.7%)	1728 (8.8%)
Heart failure	3121 (33.1%)	4473 (20.4%)	2455 (32.2%)	2070 (22.9%)

Observations represent unique patients (biological replicates); there were no technical replicates. Before PSM, the LVNC with AF and LVNC without AF cohorts included 29 356 and 39 339 patients, respectively. After PSM, 19 615 patients were included in each cohort. Values are presented as the number of patients with the outcome within 3 years, expressed as *n* (%).New-onset heart failure was analysed after excluding patients with heart failure before the index event; therefore, percentages for this outcome were calculated using the at-risk population only. Before PSM, the denominators for new-onset heart failure were 9434 in the LVNC with AF cohort and 21 878 in the LVNC without AF cohort. After PSM, the corresponding denominators were 7618 and 9,020, respectively.

PSM, propensity score matching.

After PSM, after a mean follow-up of 793.7 days (SD 421.6) in the LVNC with AF cohort and 805.7 days (SD 407.1) in the LVNC without AF cohort (median 1095 days in both, IQR 706 in LVNC with AF and 661 in LVNC without AF), the 3 year incidences were as follows: stroke in 2767 patients overall [1632 (8.3%) vs. 1135 (5.8%)], all-cause death in 7813 patients overall [4321 (22.0%) vs. 3492 (17.8%)], acute myocardial infarction in 3429 patients overall [1701 (8.7%) vs. 1728 (8.8%)], and new-onset heart failure in 4525 patients overall [2455 (32.2%) vs. 2070 (22.9%)] (*Table 2*).

#### 3.3.3 HRs before and after PSM

**Figure 2:**
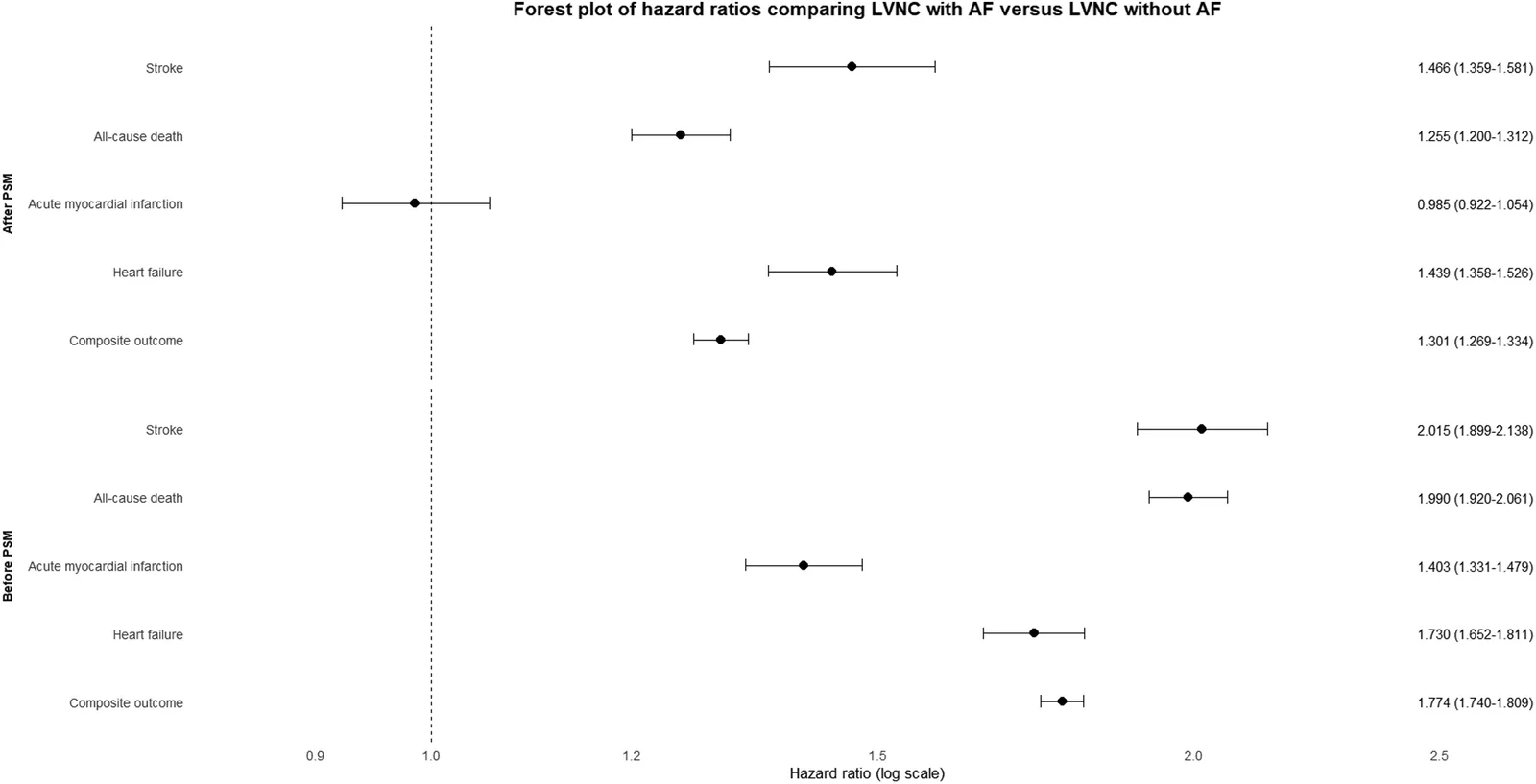
Forest plot of HRs comparing LVNC with AF vs. LVNC without AF before and after PSM. Observations represent unique patients (biological replicates); there were no technical replicates. Before PSM: HRs and 95% CIs were estimated using univariable Cox proportional hazards models.

Before PSM, patients with LVNC and AF had higher hazards of stroke (HR 2.015, 95% CI 1.899–2.138), all-cause death (HR 1.990, 95% CI 1.920–2.061), acute myocardial infarction (HR 1.403, 95% CI 1.331–1.479), new-onset heart failure (HR 1.730, 95% CI 1.652–1.811), and the composite outcome (HR 1.774, 95% CI 1.740–1.809) compared with patients with LVNC without AF (*Figure 2*).

After PSM, patients with LVNC and AF remained at higher hazard of stroke (HR 1.466, 95% CI 1.359–1.581), all-cause death (HR 1.255, 95% CI 1.200–1.312), new-onset heart failure (HR 1.439, 95% CI 1.358–1.526), and the composite outcome (HR 1.301, 95% CI 1.269–1.334), whereas the hazard of acute myocardial infarction was no longer significantly different between groups (HR 0.985, 95% CI 0.922–1.054) (*Figure 2*).

### 3.4 Multivariable Cox proportional hazards model

After adjustment, patients with LVNC and AF, compared with patients with LVNC without AF, had higher hazards of the composite outcome (HR 1.453, 95% CI 1.422–1.485), death (HR 1.660, 95% CI 1.598–1.725), stroke (HR 1.605, 95% CI 1.505–1.711), and heart failure (HR 1.512, 95% CI 1.479–1.547) (see Supplementary material online, *Table S3*).

### 3.5 Subgroup analysis

**Figure 3:**
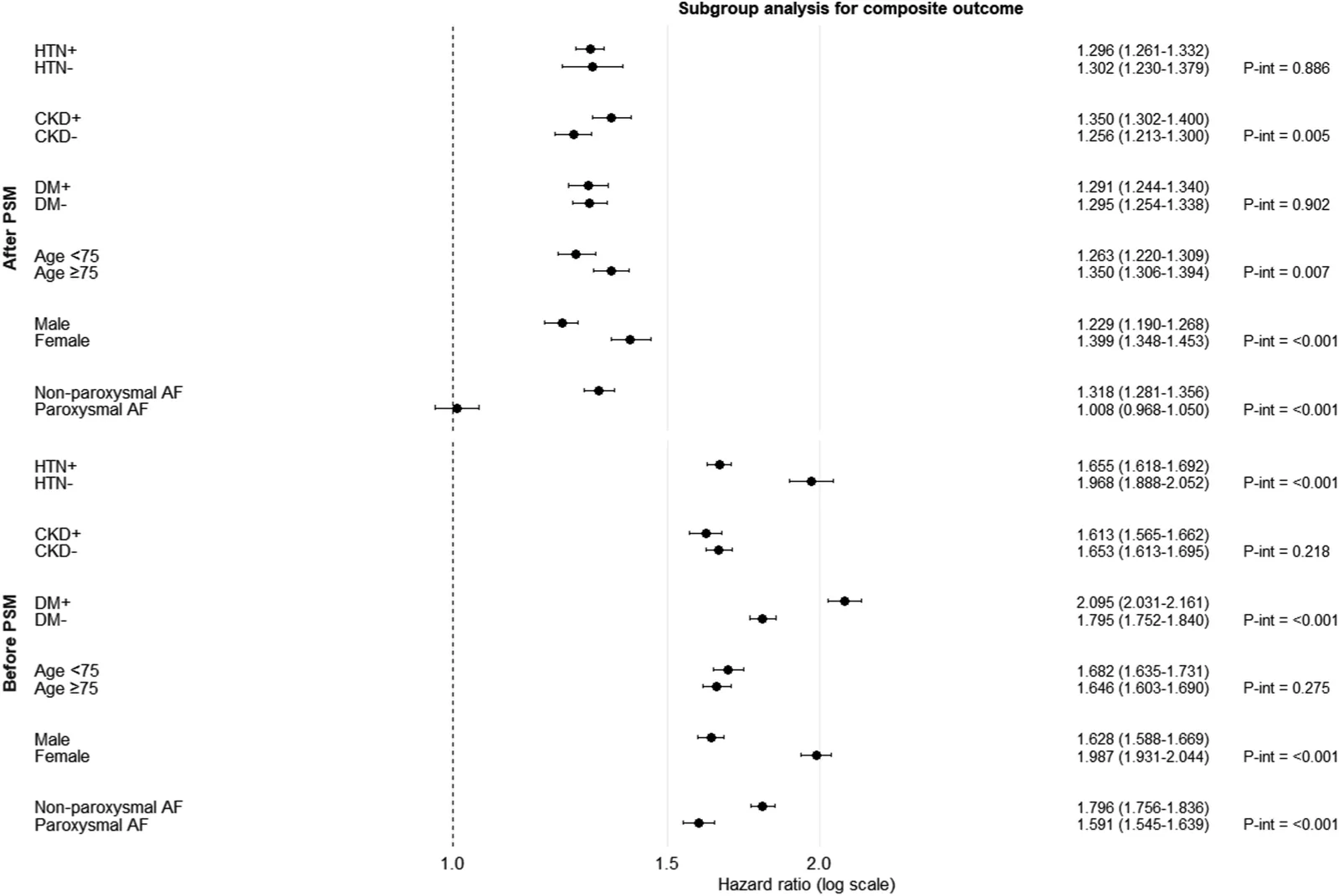
Forest plot of HRs comparing LVNC with AF vs. LVNC without AF before and after PSM for subgroup analysis. Observations represent unique patients (biological replicates); there were no technical replicates. Within each age subgroup, PSM and univariable Cox proportional hazards models were applied using the same specifications as the primary analysis.

After PSM, subgroup analyses showed findings broadly consistent with the primary analysis, with patients with LVNC and AF demonstrating a higher risk of the composite outcome across most subgroups. The association appeared more pronounced among females, patients aged ≥75 years, those with CKD, and those with non-paroxysmal AF (*Figure 3*).

### 3.6 Sensitivity analysis

#### 3.6.1 Landmark sensitivity analysis

In the sensitivity analysis evaluating outcomes occurring between 31 and 1126 days after the index event, results were consistent with the main analysis demonstrating higher risk of composite outcome (see Supplementary material online, *Table S4*).

#### 3.6.2 Echocardiography or cardiovascular magnetic resonance (CMR) imaging performed within 6 months before or after LVNC diagnosis

In two additional sensitivity analyses restricted to patients who underwent echocardiography or CMR within 6 months before or after the diagnosis of LVNC, patients with LVNC and AF consistently exhibited a higher risk of the composite outcome (see Supplementary material online, *Table S4*).

#### 3.6.3 External sensitivity analysis using the global network

In additional sensitivity analysis using data from the global network rather than the US network, the results remained consistent with the main analysis (see Supplementary material online, *Table S4*).

#### 3.6.4 Sensitivity analysis excluding death as a competing event

In an additional sensitivity analysis excluding patients who died between 1 January 2014 and 1 January 2023, thereby reducing the potential competing risk of death for the other outcomes, the results remained consistent with the main analysis (see Supplementary material online, *Table S4*).

#### 3.6.5 Comparative analysis of AF patients with LVNC versus AF patients without LVNC

After PSM, patients with AF and LVNC, compared with AF patients without LVNC, had higher risks of new-onset heart failure (HR 2.502, 95% CI 2.364–2.648), all-cause death (HR 1.109, 95% CI 1.073–1.146), and the composite outcome (HR 1.408, 95% CI 1.381–1.435) (see Supplementary material online, *Table S5*).

#### 3.6.6 Comparative analysis of AF patients with LVNC versus AF patients with Dilated cardiomyopathy (DCM)

After PSM, patients with LVNC and AF, compared with AF patients with DCM, had higher risk of all-cause death (HR 1.182, 95% CI 1.143–1.222), but lower risks of new-onset heart failure (HR 0.883, 95% CI 0.841–0.928) and the composite outcome (HR 0.863, 95% CI 0.847–0.879) (see Supplementary material online, *Table S5*).

#### 3.6.7 Comparative analysis of AF patients with LVNC versus AF patients with hypertrophic cardiomyopathy (HCM)

After PSM, patients with LVNC and AF, compared with AF patients with HCM, had lower risks of stroke (HR 0.880, 95% CI 0.804–0.963) and acute myocardial infarction (HR 0.811, 95% CI 0.745–0.883), but higher risks of new-onset heart failure (HR 1.455, 95% CI 1.338–1.583) and the composite outcome (HR 1.122, 95% CI 1.084–1.161) (see Supplementary material online, *Table S5*).

#### 3.6.8 Comparative analysis of AF patients with LVNC versus AF patients without cardiomyopathy

After PSM, patients with LVNC and AF compared with AF patients without cardiomyopathy had higher risks of new-onset heart failure (HR 2.403, 95% CI 2.236–2.584), all-cause death (HR 1.055, 95% CI 1.010–1.102), and the composite outcome (HR 1.368, 95% CI 1.333–1.403) (see Supplementary material online, *Table S5*).

## 4. Discussion

To our knowledge, this study represents one of the largest cohorts of patients with LVNC evaluated in relation to AF and AF-related outcomes. The principal findings of our study are as follows: (i) patients with LVNC and AF were older, with a more comorbid profile, compared with patients with LVNC without AF; (ii) the 1 year incidence of AF in patients with LVNC was 36 per 1000 person-years; (iii) patients with LVNC and AF were found to have higher rates of all-cause death, stroke, and new-onset heart failure compared with patients with LVNC without AF; and (iv) a more pronounced increase in the risk of the composite outcome was observed in patients with CKD, those aged ≥75 years, females, and those with non-paroxysmal AF.

In agreement with our findings regarding the more comorbid clinical phenotype of patients with AF and LVNC, Stöllberger et al.^8^ described a cohort of 102 LVNC patients, whereby patients with LVNC and AF (*n* = 15) were older and had more heart failure and diabetes. Comorbidities such as hypertension, diabetes, CKD, and dyslipidaemia are an integral element of the AF patient phenotype, particularly in the elderly.^10,11^ Thus, patients with LVNC and AF commonly present with multiple comorbidities, which may contribute to their higher observed risk of adverse clinical events, underscoring the importance of optimal management of comorbidities in this high-risk phenotype.

In our study, the incidence of AF was 36 per 1000 person-years in patients with LVNC, which is substantially higher than in the general population. For example, the analysis by Wu et al.^12^ of 3.4 million individuals reported a standardized AF incidence that increased by 30% comparing 2017 and 1998 from 2.47 to 3.22 per 1000 person-years, respectively.

The higher incidence of AF in patients with LVNC may be related to structural cardiac remodelling and other disease-related factors that predispose to atrial arrhythmogenesis. Prior studies have suggested that genetic factors may also contribute to the coexistence of LVNC and AF.^13–15^ Additionally, mutations in the HCN4 gene have been associated with early onset and frequent occurrence of AF and LVNC.^16^ However, our study did not include genotype data, and therefore no mechanistic inference regarding genetic predisposition can be made from the present analysis. Further genotype-phenotype studies in larger cohorts are needed to clarify the mechanisms underlying this association. The observed incidence of new-onset AF in patients with LVNC suggests that closer clinical follow-up may be warranted and that further studies are needed to clarify whether targeted screening strategies may be beneficial.

We found more pronounced increase in composite risk among patients with CKD, those aged ≥75 years, and those with non-paroxysmal AF highlighting a high-risk clinical phenotype in patients with LVNC and AF. AF is commonly associated with older age and comorbidities such as CKD, reflecting a state of clinical complexity that is associated with a higher risk of adverse events.^10,11^ Analysis of 29 181 patients from the Global Anticoagulant Registry in the FIELD-Atrial Fibrillation demonstrated that non-paroxysmal AF patterns were associated with higher risks of stroke/systemic embolism, new or worsening heart failure, and death than paroxysmal AF.^17^ Overall, these subgroup findings suggest that AF-related risk in LVNC is heterogeneous and may be greatest in older patients with a greater comorbidity burden and more persistent forms of AF.

Although a higher risk of adverse clinical outcomes, such as thromboembolic events, in patients with LVNC has been reported in recent studies,^15,18^ there are limited data regarding the clinical outcomes of patients with AF and LVNC.^8^ In the first Zurich cohort of 34 patients with LVNC, AF was seen in 25%.^18^ Stöllberger et al.^8^ followed a small cohort of 102 patients with LVNC for a mean of 3.8 years, reporting higher mortality in the 15 patients (15%) with LVNC who had AF. Our findings extend the limited available evidence by evaluating a substantially larger cohort of patients with LVNC. Taken together, these observations suggest that a holistic or integrated management approach may be relevant in patients with LVNC and AF. This strategy is implemented in the *Atrial Fibrillation Better Care* (ABC) pathway, which is supported by randomized trials and observational cohort studies.^19–21^ Newer variants of the ABC acronym (untested in trials) have been introduced in the 2023 American College of Cardiology/American Heart Association/American College of Clinical Pharmacy/Heart Rhythm Society guidelines (as ‘stroke prevention, optimization of comorbidities, and symptom management’) and in the 2024 ESC guidelines (as ‘CARE’, i.e. Comorbidity and risk factors, Avoid stroke and thromboembolism, Reduce symptoms and Evaluation and dynamic reassessment).^22,23^

## 5. Limitations

Our study has some limitations. First, the diagnosis of LVNC was based on ICD-10 coding, which may not perfectly capture all true cases and could lead to misclassification. Also, the definition of LVNC has not been standardized beyond the ICD-10 coding.^24^ Second, the retrospective, observational design is subject to selection bias and allows only the identification of associations, rather than establishing causal relationships. Third, although we included multiple covariates, including demographics, ethnicity, baseline clinical characteristics, and medications, in the propensity score model to minimize confounding, residual confounding due to unmeasured variables may still be present. Fourth, although we did PSM to reduce the absolute mean differences between the two cohorts, residual imbalance remained in BMI after matching, which may have influenced the results. Fifth, LVNC was identified solely on the basis of ICD 10 codes, without confirmation using imaging criteria, which may have introduced substantial misclassification bias and influenced the results of our study. Furthermore, changing nomenclature and variability in the description of LVNC may also have affected case ascertainment. Despite additional multivariable Cox regression, broader PSM, and multiple sensitivity analyses, residual and unmeasured confounding cannot be fully excluded in this observational study. Sixth, subgroup analyses were exploratory and based on the same overall modelling framework as the primary analysis; therefore, they should be interpreted cautiously and not as resolving residual confounding. Seventh, no formal imputation of missing data was performed in TriNetX. Eighth, analyses in TriNetX relied on the platform’s built-in greedy nearest-neighbour PSM approach with a default calliper width of 0.1 pooled standard deviations, and alternative matching specifications or causal inference methods, including inverse probability of treatment weighting, could not be implemented within the analytic workflow used in this study. Ninth, although the findings of the sensitivity analysis using the global network were consistent with those of the primary analysis conducted in the US network, the generalizability of our results to other healthcare settings not represented within the TriNetX network remains limited.

## 6. Conclusion

Although LVNC is associated with a high incidence of AF and an increased thromboembolic risk, the presence of AF in patients with LVNC further amplifies the risks of stroke, myocardial infarction, heart failure, and mortality.

## Supplementary Material

cvag156_Supplementary_Data

## Data Availability

Data used in this research project will be available upon a reasonable request from the corresponding authors.
