# Supplementary material for: Atrial fibrillation in patients with left ventricular non-compaction: incidence and associations with stroke, heart failure, and mortality
Source: Cardiovasc Res. 2026 Jul 9;122(13):1761–9. doi: 10.1093/cvr/cvag156 (PMC13604827; doi:10.1093/cvr/cvag156)
Supplement: Supplementary file 1 [file cvag156_supplementary_data.zip › Supplementary materials (10).docx]

**Supplementary materials**

**Atrial fibrillation in Left ventricular non-compaction: global real-world analysis from incidence to stroke, heart failure, and mortality**

**TriNetX Database**

The TriNetX data are collected from member healthcare organizations (HCO) and originates from their primary electronic health records (EHR) system. A typical HCO is a large academic health centre with data coming from majority of its affiliates. A single HCO frequently has more than one facility, including main and satellite hospitals. The data are stored on the TriNetX database via a physical server at the institution’s data centre or a virtual hosted appliance. The TriNetX platform comprises of a series of these appliances connected into a federated network. This network can broadcast queries to each appliance. Results are subsequently collected and aggregated. Once the data are sent to the network, it is mapped to a standard and controlled set of clinical terminologies and undergoes a data quality assessment including ‘data cleaning’ that rejects records which do not meet the TriNetX quality standards. The TriNetX database performs internal and extensive data quality assessment with every refresh based on conformance, completeness, and plausibility (http://doi.org/10.13063/2327-9214.1244). HIPAA (Health Insurance Portability and Accountability Act) compliance of the clinical patient data is achieved using deidentification. Available data types within the network include demographics, diagnoses (represented by ICD-10-CM codes), procedures (coded in ICD-10-PCS or CPT), and measurements (coded to LOINC). While extensive information is provided about patients’ diagnoses and procedures, other variables (such as socioeconomic and lifetime factors are not comprehensively represented). The advantage of EHR data over insurance claim data is that both insured and uninsured patients are included. An advantage of EHR data over survey data is that the former represents the diagnostic rates in the population presenting to healthcare facilities. This provides an accurate account of the burden of specific diagnoses on healthcare systems. One primary limitation of relying on diagnoses is that they do not account for undiagnosed patients who might have a condition but have not yet received medical support. Another general limitation of EHR data is that a patient may be seen in different HCO for different components of their care. If one HCO is not part of the federated network, then part of their medical records may not be available. Using a network of healthcare organizations, rather than a single site, limits this possibility but does not fully remove it.

Propensity Score Matched Analyses were performed using logistic regression [Logistic Regression from the scikit-learn package in Python (version 3.7)]. TriNetX performed a 1:1 greedy nearest neighbour matching model with a calliper of 0.1 pooled standard deviations. To eliminate bias resulting from nearest neighbours’ algorithms, the rows were randomized. Any baseline characteristic with a standardized mean difference between cohorts lower than 0.1 was deemed well matched.

**Assessment of the Proportional Hazards Assumption**

To evaluate whether the proportional hazards assumption was satisfied in the Cox regression models, we conducted a Chi-square (χ²) test based on Schoenfeld residuals. These tests examine whether the relationship between the associated variables and the hazard function remains stable over time. The null hypothesis posits that the effect of OAC discontinuation on the hazards of primary outcomes is constant throughout the study period. The χ² statistic measures the discrepancy between the observed and expected Schoenfeld residuals. A higher χ² value indicates a greater divergence from the expected values, suggesting a potential violation of the proportional hazards’ assumption. Conversely, a lower χ² value implies that the observed residuals closely align with the expected values, supporting the assumption. The p-value, derived from the χ² statistic, reflects the likelihood of observing these deviations under the null hypothesis. A p-value greater than 0.05 suggests that the deviations are likely due to random variation, indicating that the proportional hazards assumption holds. In contrast, a p-value less than 0.05 implies that the observed deviations are unlikely to be random, indicating a violation of the proportional hazards’ assumption.

**Supplementary Table 1. One-year incidence of AF in patients with LVNC according to age, sex, and ethnicity.**

| **Category** | **Incidence proportion (%)** | **Incidence rate (per 1000 PY)** |
| --- | --- | --- |
| Overall (2023-01-01 to 2024-01-01) | 3.377 | 36.065 |
| **Age band** |  |  |
| 15-19 | 4.202 | 44.871 |
| 20-24 | 4.016 | 44.142 |
| 25-29 | 2.762 | 29.697 |
| 30-34 | 1.880 | 19.781 |
| 35-39 | 1.460 | 15.239 |
| 40-44 | 2.051 | 21.818 |
| 45-49 | 1.168 | 12.312 |
| 50-54 | 1.896 | 19.804 |
| 55-59 | 2.757 | 29.202 |
| 60-64 | 3.741 | 39.478 |
| 65-69 | 3.415 | 36.315 |
| 70-74 | 4.306 | 46.006 |
| 75-79 | 5.015 | 54.289 |
| 80-84 | 4.010 | 43.466 |
| 85+ | 8.174 | 94.868 |
| **Sex** |  |  |
| Female | 2.638 | 27.990 |
| Male | 4.108 | 44.142 |
| **Ethnicity** |  |  |
| Hispanic or Latino | 2.486 | 26.783 |
| Not Hispanic or Latino | 3.364 | 35.762 |
| Unknown | 3.580 | 38.606 |

Values were transcribed from the uploaded TriNetX incidence and prevalence analysis report for the US Collaborative Network. The table reflects the cohort defined in that report as adults with LVNC, excluding ischemic cardiomyopathy, dilated cardiomyopathy, obstructive hypertrophic cardiomyopathy, other hypertrophic cardiomyopathy, and alcoholic cardiomyopathy, and without prior atrial fibrillation or flutter before the start of the time window. The report cohort included 39,339 patients. Incidence proportion is presented as a percentage. Incidence rates were converted from the report’s cases/person-day metric to events per 1,000 person-years using: incidence rate × 365.25 × 1,000. No hypothesis testing was performed for this descriptive table; therefore, no P values are reported.

**Supplementary Table 2. Overall frequency of treatment pathways and percentage distribution across lines of treatment in patients with LVNC and AF**.

| **Treatment pathway** | **Overall, n (%)** | **LOT1, %** | **LOT2, %** | **LOT3, %** | **LOT4, %** | **LOT5, %** | **LOT6, %** |
| --- | --- | --- | --- | --- | --- | --- | --- |
| Beta blockers/related | 11,884 (46.50) | 28.47 | 17.83 | 22.19 | 21.91 | 23.12 | 23.11 |
| Anticoagulants | 11,024 (43.13) | 24.94 | 17.78 | 20.77 | 21.51 | 23.12 | 23.52 |
| Beta blockers/related + Anticoagulants | 10,708 (41.90) | 18.66 | 18.13 | 16.06 | 14.83 | 14.61 | 14.47 |
| Antiarrhythmics | 7,719 (30.20) | 11.31 | 16.82 | 13.85 | 16.20 | 16.00 | 17.10 |
| Beta blockers/related + Antiarrhythmics + Anticoagulants | 6,190 (24.22) | 6.32 | 10.39 | 11.07 | 9.57 | 8.45 | 7.93 |
| Antiarrhythmics + Anticoagulants | 4,982 (19.49) | 5.72 | 10.27 | 8.02 | 7.79 | 7.69 | 7.15 |
| Beta blockers/related + Antiarrhythmics | 3,578 (14.00) | 3.77 | 6.97 | 6.20 | 6.63 | 5.61 | 5.61 |
| Beta blockers/related + Antiarrhythmics + Anticoagulants + Catheter ablation | 324 (1.27) | 0.26 | 0.51 | 0.73 | 0.50 | 0.43 | 0.38 |
| Antiarrhythmics + Anticoagulants + Catheter ablation | 239 (0.94) | 0.22 | 0.61 | 0.60 | 0.44 | 0.38 | 0.48 |
| Anticoagulants + Catheter ablation | 102 (0.40) | 0.12 | 0.42 | 0.30 | 0.26 | 0.34 | 0.15 |
| Beta blockers/related + Anticoagulants + Catheter ablation | 70 (0.27) | 0.08 | 0.16 | 0.09 | 0.13 | 0.07 | 0.10 |
| Catheter ablation | 45 (0.18) | 0.07 | 0.06 | 0.05 | 0.14 | 0.05 | 0.00 |
| Antiarrhythmics + Catheter ablation | 22 (0.09) | 0.04 | 0.02 | 0.03 | 0.06 | 0.02 | 0.00 |
| Beta blockers/related + Catheter ablation | 14 (0.05) | 0.01 | 0.03 | 0.03 | 0.01 | 0.04 | 0.00 |
| Beta blockers/related + Antiarrhythmics + Catheter ablation | 10 (0.04) | 0.00 | 0.01 | 0.03 | 0.01 | 0.05 | 0.00 |

AF, atrial fibrillation; LOT, line of treatment; LVNC, left ventricular non-compaction. LOT1 to LOT6 represent sequential treatment lines identified by the treatment pathways analysis. LOT1 corresponds to the initial treatment regimen, and each subsequent LOT reflects a newly observed regimen after completion or modification of the prior l

**Supplementary Table 3. Multivariable Cox proportional hazards model results across study outcomes**

| **Predictor** | **Stroke**  **aHR (95% CI)** | **Heart failure**  **aHR (95% CI)** | **AMI**  **aHR (95% CI)** | **Death**  **aHR (95% CI)** | **Composite outcome**  **aHR (95% CI)** |
| --- | --- | --- | --- | --- | --- |
| LVNC with AF compared to LVNC without AF | 1.605 (1.505-1.711) | 1.512 (1.479-1.547) | 1.038 (0.980-1.100) | 1.660 (1.598-1.725) | 1.453 (1.422-1.485) |
| Hispanic or Latino | 1.025 (0.889-1.182) | 1.032 (0.983-1.082) | 0.894 (0.779-1.027) | 0.747 (0.678-0.822) | 1.001 (0.957-1.047) |
| White | 1.047 (0.941-1.164) | 0.959 (0.927-0.992) | 1.466 (1.319-1.629) | 1.113 (1.045-1.186) | 0.967 (0.936-0.998) |
| Asian | 1.209 (0.933-1.565) | 0.971 (0.885-1.066) | 1.315 (1.028-1.682) | 0.765 (0.642-0.910) | 0.983 (0.901-1.072) |
| Black or African American | 1.454 (1.296-1.632) | 1.270 (1.223-1.320) | 1.587 (1.414-1.782) | 1.138 (1.060-1.221) | 1.231 (1.187-1.277) |
| Hypertensive diseases | 1.117 (1.026-1.215) | 0.914 (0.889-0.939) | 0.943 (0.875-1.016) | 0.916 (0.874-0.961) | 0.921 (0.897-0.946) |
| Ischemic heart diseases | 1.035 (0.967-1.107) | 0.938 (0.916-0.961) | 2.146 (2.015-2.286) | 1.150 (1.104-1.199) | 1.020 (0.997-1.044) |
| Peripheral artery disease | 1.157 (1.058-1.264) | 1.087 (1.047-1.128) | 1.197 (1.102-1.301) | 1.227 (1.160-1.297) | 1.087 (1.049-1.125) |
| Cerebral infarction | 12.042 (11.278-12.857) | 0.996 (0.957-1.037) | 1.309 (1.198-1.430) | 1.199 (1.127-1.275) | 1.403 (1.354-1.455) |
| Diabetes mellitus | 1.159 (1.082-1.242) | 1.084 (1.057-1.112) | 1.168 (1.096-1.245) | 1.287 (1.233-1.343) | 1.106 (1.079-1.133) |
| Dyslipidaemia | 1.056 (0.982-1.134) | 0.888 (0.866-0.910) | 0.954 (0.894-1.018) | 0.786 (0.754-0.821) | 0.892 (0.871-0.913) |
| Heart failure | 0.890 (0.831-0.954) | 2.646 (2.580-2.713) | 0.955 (0.896-1.018) | 1.394 (1.336-1.455) | 2.136 (2.086-2.187) |
| COPD | 1.014 (0.934-1.100) | 1.228 (1.193-1.265) | 1.197 (1.115-1.285) | 1.500 (1.434-1.570) | 1.237 (1.203-1.272) |
| CKD | 1.051 (0.977-1.129) | 1.283 (1.249-1.317) | 1.350 (1.265-1.442) | 1.831 (1.755-1.911) | 1.289 (1.257-1.322) |
| Neoplasms | 0.891 (0.835-0.951) | 0.989 (0.966-1.012) | 1.017 (0.960-1.078) | 1.249 (1.203-1.298) | 1.024 (1.001-1.046) |
| Beta blockers/related | 0.963 (0.886-1.048) | 0.848 (0.824-0.872) | 0.873 (0.810-0.941) | 0.733 (0.698-0.769) | 0.864 (0.841-0.888) |
| Diuretics | 0.955 (0.884-1.033) | 1.458 (1.417-1.499) | 0.935 (0.871-1.004) | 1.283 (1.221-1.347) | 1.393 (1.356-1.430) |
| Antiarrhythmics | 0.956 (0.893-1.024) | 1.001 (0.977-1.026) | 1.034 (0.971-1.101) | 0.994 (0.954-1.036) | 1.002 (0.979-1.025) |
| Calcium channel blockers | 1.110 (1.036-1.190) | 0.958 (0.934-0.982) | 1.135 (1.066-1.210) | 0.867 (0.830-0.905) | 0.975 (0.952-0.999) |
| ACE inhibitors | 0.996 (0.931-1.065) | 0.950 (0.928-0.973) | 1.007 (0.947-1.070) | 0.806 (0.774-0.840) | 0.951 (0.930-0.973) |
| Anticoagulants | 0.988 (0.913-1.070) | 0.965 (0.939-0.991) | 1.400 (1.302-1.505) | 1.120 (1.068-1.175) | 1.033 (1.006-1.060) |
| LVEF (%) | 0.997 (0.989-1.005) | 0.989 (0.986-0.992) | 1.003 (0.995-1.010) | 0.996 (0.992-1.001) | 0.991 (0.988-0.994) |
| BMI (kg/m2) | 0.988 (0.983-0.992) | 1.003 (1.002-1.005) | 0.991 (0.987-0.995) | 0.968 (0.965-0.971) | 0.999 (0.998-1.001) |

Values are presented as hazard ratios (HRs) with 95% confidence intervals (CIs). AMI = acute myocardial infarction. The composite outcome comprised all-cause death, stroke, acute myocardial infarction, and heart failure.

**Supplementary Table 4. Sensitivity analyses comparing patients with LVNC and AF versus patients with LVNC without AF**

| **Sensitivity analysis** | **Analysis** | **Composite outcome HR (95% CI)** | **Stroke HR (95% CI)** | **AMI HR (95% CI)** | **New-onset HF HR (95% CI)** | **All-cause death HR (95% CI)** |
| --- | --- | --- | --- | --- | --- | --- |
| **Landmark analysis (31-1126 days after index event)** | Before PSM | 2.057 (1.928-2.194) | 1.477 (1.390-1.570) | 1.784 (1.694-1.879) | 1.946 (1.872-2.023) | 1.768 (1.732-1.805) |
|  | After PSM | 1.408 (1.297-1.528) | 0.975 (0.903-1.053) | 1.415 (1.324-1.512) | 1.215 (1.157-1.276) | 1.256 (1.223-1.290) |
| **Echocardiography within +/- 6 months of LVNC diagnosis** | Before PSM | 1.683 (1.640-1.727) | 1.830 (1.693-1.978) | 1.250 (1.168-1.338) | 1.719 (1.599-1.848) | 1.944 (1.854-2.038) |
|  | After PSM | 1.245 (1.204-1.287) | 1.266 (1.147-1.398) | 1.092 (0.957-1.245) | 1.345 (1.224-1.477) | 1.267 (1.194-1.345) |
| **CMR within +/- 6 months of LVNC diagnosis** | Before PSM | 1.710 (1.547-1.891) | 2.054 (1.441-2.929) | 1.344 (1.012-1.785) | 1.540 (1.198-1.980) | 1.829 (1.463-2.288) |
|  | After PSM | 1.194 (1.046-1.363) | 1.309 (0.854-2.006) | 0.816 (0.578-1.150) | 1.162 (0.830-1.627) | 1.184 (0.885-1.584) |
| **Global collaborative network** | Before PSM | 1.762 (1.730-1.794) | 2.020 (1.909-2.138) | 1.406 (1.336-1.481) | 1.693 (1.621-1.768) | 1.974 (1.908-2.042) |
|  | After PSM | 1.306 (1.275-1.337) | 1.432 (1.332-1.541) | 0.987 (0.924-1.053) | 1.463 (1.383-1.547) | 1.235 (1.183-1.289) |
| **Excluding death before index event** | Before PSM | 1.695 (1.656-1.735) | 2.011 (1.876-2.156) | 1.343 (1.260-1.431) | 1.623 (1.543-1.708) | 3.654 (3.092-4.317) |
|  | After PSM | 1.307 (1.268-1.347) | 1.422 (1.299-1.556) | 0.928 (0.855-1.006) | 1.395 (1.305-1.491) | 1.248 (1.007-1.547) |

AF, atrial fibrillation; AMI, acute myocardial infarction; CI, confidence interval; CMR, cardiac magnetic resonance; HF, heart failure; HR, hazard ratio; LVNC, left ventricular non-compaction; PSM, propensity score matching.

**Supplementary Table 5. Comparative Analysis of AF Patients with LVNC versus other cohorts**

| **Comparator AF cohort** | **Analysis** | **Composite outcome HR (95% CI)** | **Stroke HR (95% CI)** | **AMI HR (95% CI)** | **New-onset HF HR (95% CI)** | **All-cause death HR (95% CI)** |
| --- | --- | --- | --- | --- | --- | --- |
| **Without LVNC** | Before PSM | 1.745 (1.727–1.764) | 1.021 (0.984–1.060) | 1.470 (1.416–1.525) | 2.492 (2.404–2.584) | 1.437 (1.404–1.470) |
|  | After PSM | 1.408 (1.381–1.435) | 0.964 (0.917–1.014) | 0.977 (0.927–1.029) | 2.502 (2.364–2.648) | 1.109 (1.073–1.146) |
| **DCM** | Before PSM | 0.900 (0.882–0.918) | 1.034 (0.977–1.094) | 1.022 (0.965–1.082) | 0.886 (0.837–0.937) | 1.207 (1.164–1.252) |
|  | After PSM | 0.863 (0.847–0.879) | 1.022 (0.969–1.078) | 1.045 (0.991–1.102) | 0.883 (0.841–0.928) | 1.182 (1.143–1.222) |
| **HCM** | Before PSM | 1.404 (1.376–1.433) | 0.826 (0.785–0.870) | 0.787 (0.748–0.828) | 1.465 (1.397–1.535) | 1.425 (1.374–1.477) |
|  | After PSM | 1.122 (1.084–1.161) | 0.880 (0.804–0.963) | 0.811 (0.745–0.883) | 1.455 (1.338–1.583) | 1.048 (0.985–1.115) |
| **Without cardiomyopathy** | Before PSM | 2.244 (2.215-2.274) | 1.022 (0.984-1.060) | 1.452 (1.399-1.507) | 2.281 (2.205-2.360) | 1.445 (1.413-1.479) |
|  | After PSM | 1.368 (1.333-1.403) | 0.939 (0.876-1.006) | 0.943 (0.882-1.009) | 2.403 (2.236-2.584) | 1.055 (1.010-1.102) |

AF, atrial fibrillation; AMI, acute myocardial infarction; DCM, dilated cardiomyopathy; HF, heart failure; HCM, hypertrophic cardiomyopathy; HR, hazard ratio; LVNC, left ventricular non-compaction; PSM, propensity score matching.

**Supplementary Figure Legends**

**Supplementary Figure 1. Study flowchart.**

All counts represent unique patients (biological replicates); there were no technical replicates.

**Supplementary Figure 2. Treatment pathways and distribution of treatment use across lines of treatment in patients with LVNC and AF.**

Panel A shows the proportion of patients receiving each treatment pathway during follow-up. Panel B shows the distribution of treatment use across successive lines of treatment (LOT1 to LOT6), expressed as the percentage of patients within each line. BB: Betablocker, OAC: oral anticoagulant, AA: Antiarrhythmic, PVI: pulmonary vein isolation.
